# Correction: Collaboration in the time of COVID: a scientometric analysis of multidisciplinary SARS-CoV-2 research

**DOI:** 10.1057/s41599-021-00957-w

**Published:** 2021-11-08

**Authors:** Eoghan Cunningham, Barry Smyth, Derek Greene

**Affiliations:** 1 Insight SFI Research Centre for Data Analytics, University College, Dublin, Ireland; 2 School of Computer Science, University College, Dublin, Ireland

**Keywords:** Science, technology and society, Complex networks

Correction to: *Humanities and Social Sciences Communications* 10.1057/s41599-021-00922-7, published online 19 October 2021.

This paper wrongly included two files among the supplementary information that were incorrectly uploaded. These files, named ‘Marked-up Manuscript’ and ‘Field of Study Networks’ have now been removed.

One correct supplementary file remains.

